# Modelling of transverse vibration of conveyor belt in aspect of the trough angle

**DOI:** 10.1038/s41598-023-46534-w

**Published:** 2023-11-14

**Authors:** Piotr Bortnowski, Robert Król, Maksymilian Ozdoba

**Affiliations:** Mining and Geology Department of Mining, Faculty of Geoengineering, Wrocław University of Science and Technology, Na Grobli 15, 50 - 421 Wrocław, Poland

**Keywords:** Mechanical engineering, Engineering, Electrical and electronic engineering

## Abstract

The transverse vibration of conveyor belts is a crucial aspect in their proper and safe design, as the correct determination of vibration frequencies is essential to avoid unsafe operating conditions and premature wear of components. This study presents the Moving Compressed Beam (MCB) transverse vibration model, that is based on the interpretation of a conveyor belt as a beam known from the literature. This approach allows you to include in the model the transverse flexural rigidity of a troughed belt-a parameter that is closely related to the belt geometry. While not commonly used in engineering practice, the MSB model was compared with other models during the laboratory tests based on changing of trough geometry. This study emphasizes the significance of considering the transverse flexural rigidity of conveyor belts during the design process, and the MCB model which offers a promising approach in aspect of vibration control.

## Introduction

Vibrations of conveyor belt are a common dynamic phenomenon that occurs during the normal operation of the conveyor^1^. The vibration characteristic is extremely important factor for the proper and uninterrupted operation of the conveyor^2,3^, and incorrect vibration conditions can lead to failure^4,5^. These vibrations can be classified as longitudinal or transverse^6,7^, depending on their direction relative to the conveyor belt. The longitudinal vibrations of the belt are an extensive research issue^8–10^ and are related to the propagation and damping of stress waves in the conveyor belt^11^. The transverse vibrations referred to in the article are cyclic displacements of the belt in the vertical direction that occur between the idler sets. These vibrations are particularly dangerous due to the possibility of the belt conveyor entering into resonance^12^. The vibrations are also directly related to the noise generated by the conveyor^13^. In belt conveyors, resonance often occurs when the natural frequency of the conveyor structure coincides with the frequency of transverse vibration in the conveyor belt^14^. This phenomenon can lead to a dangerous increase in vibration amplitudes, which can cause damage to the conveyor system and its components^15^. Roller bearings are especially vulnerable to resonances, as excessive vibrations can cause premature wear and damage to the bearings^16^. In extreme cases, resonance can cause complete failure of the conveyor system, resulting in significant repair costs^17^, loss of productivity^18^, and posing a serious safety hazard to personnel and equipment^19^.

Given the potential risks associated with resonance in conveyor systems, it is crucial to mitigate and identify its causes^20^. This can be achieved by implementing appropriate design measures during the conveyor belt design phase and conducting regular maintenance and monitoring during operation. Preventing resonance in conveyor systems during the design stage primarily involves using appropriate models that can predict transverse vibrations based on assumed operating parameters^21^. However, there are also vibrations induced in an uncontrolled manner, which can result from various factors, such as the construction of the belt^22^, electromechanical operating parameters^23^ and manufacturing defects^24^. The second group of vibrations is associated with vibrations generated during the operation of the conveyor as a result of changing operating parameters or intentionally inducing vibrations^25^. Many of these vibration groups cannot be included in the calculations due to their non-stationary nature. Several publications have highlighted the dominant importance of eccentricity and unbalance of the rollers^26^ in causing resonance in conveyor systems. The frequency of vibration resulting from the rotation of the idlers is a key factor^27^ considered during the conveyor securing stage. The design process typically focuses on ensuring proper spacing of idler supports and determining the tensile force in the belt^28^, as these parameters significantly affect the level of vibrations generated. The aforementioned parameters can be duly incorporated and validated via an apt predictive model.

The article presents a model based on the moving compressed beam (MCB), which allows for the prediction of the transverse vibration frequency of the conveyor belt. This model consider various factors such as the geometry of the cross-section of the conveyor belt, Young’s modulus, and the dynamic of the belt. The MCB model is an advanced design approach for belt conveyors that considers the transverse flexural rigidity of the belt, specifically accounting the shape of the belt. The model recognizes that the flexural rigidity increases as the trough angle of the belt increases, providing a more comprehensive understanding of belt conveyor behavior.The article provides an in-depth analysis of currently used forecast models, including their practical advantages and disadvantages. Theoretical assumptions of the MCB model based on Bernoulli-Euler beam are then presented, along with a full interpretation and modifications. In the following stage, the sensitivity of the model to all input parameters is analyzed, with a particular focus on the impact of flexural rigidity on the results. The model is then validated in laboratory conditions, where actual values of transverse vibrations of the moving belt are measured using a mobile device placed on the conveyor belt. The results are discussed and compared with the predictions of discussed theoretical models.

## The prior art

Belt vibration models are generally classified into two types: static and dynamic^29^. In static models, the main assumption for solving the equations is that the system is in equilibrium and there are no vibrations^30^. Such models are used to determine the tensioning forces of the belt^31^, calculate deflection, and determine the frequency of natural vibrations^32^. The external load on the belt is taken into consideration, and boundary conditions are determined. In contrast, dynamic models consider the movement and vibration of the belt, and are used to study the belt’s dynamic behavior under different operating conditions^33^. The belt is treated as a continuous elastic system subject to complex forces in different directions. Discrete elements into which the belt is divided^34^ or a small section of the belt can be modeled as a continuous system^35^. Static models are simpler to use than dynamic models, but do not consider the dynamic behavior of the belt. Dynamic models, on the other hand, provide more detailed information about the motion of the belt, but are more complicated to use. It’s worth noting that many of the currently used models are static models modified with motion parameters, making the concept of “static” in such systems vague.

The string model is a commonly used computational model for predicting transverse vibrations in belts^36^. It is assumed in the calculations that the belt behaves like a perfectly flexible string, transmitting longitudinal forces but experiencing no bending stresses^37^. The wave equation relates the transverse displacement to the wave propagation speed, tension, and linear mass density of the string^38^. In most models, it is assumed that the vibrating string is homogeneous, and that the cross-sectional area and density are constant along the length of the string. By making this assumption, a single value for the linear mass density of the string can be used in the wave equation, thereby simplifying the analysis. However, if the string exhibits non-homogeneity, this assumption may require reconsideration, leading to necessary modifications in the analysis^39^. The string model is particularly developed for belt gears, and many of them consider nonlinear material parameters, damping and belt speed^6^. Some of them also take into account long-term changes in the speed of the belt^40^. The main disadvantage of string models is that they neglect the bending stiffness of the cross-section and the bending moments that occur in a loaded conveyor belt. Prior research^41,42^ indicates that this parameter exerts a substantial influence on the transverse vibrations of the belts. Hence, these models do not always allow for a correct prediction.

A beam model is a more advanced approach for analyzing the transverse vibrations of a belt, as it takes into account the bending stiffness of the belt cross-section and the bending moments that occur in a loaded conveyor belt^43^. The model presume that the material will undergo bending when subjected to a load, which is why the term flexural rigidity is employed. In this model, the transverse displacement of the beam is described by a equation that takes into account the bending moment and shear force acting on the beam, as well as the transverse loading applied to the beam^44^. The flexural rigidity of the beam, which is a function of the cross-sectional area and material properties, determines how the beam resists bending deformation. Conveyor belts are subject to the influence of both transverse and longitudinal stiffness, which have a significant impact on their behavior^45,46^. In particular, transverse stiffness governs the belt’s ability to be troughed^47,48^. The standard beam models used to analyze the transverse vibrations of a conveyor belt have a significant drawback, namely, they do not take into account the longitudinal stretching forces that are present in the belt during normal operation. These forces are crucial for ensuring the proper functioning of the conveyor under steady state conditions^49^, and their omission can lead to inaccurate results. Therefore, more advanced beam models that incorporate longitudinal stretching forces should be considered in order to obtain a more accurate representation of the belt’s behavior.

Lodewijks proposed a solution^50^ to the problem of accurately modeling conveyor belt vibrations by developing a specific model for this purpose. The model takes into account various parameters such as longitudinal tensile forces, belt speed and stress wave speed, transverse displacement of the belt or the influence of the load distributed on the belt on its deflection. The model is based on a set of partial differential equations that describe the motion of the belt, including both longitudinal and transverse vibrations. The equations are solved numerically using finite difference method (FEM). In this model, the belt is assumed to be a thin and flexible beam, and the transverse vibrations of the belt are modeled as standing waves. Lodewijsk presents a solution under the following assumptions: the cross-section rigidity of the conveyor belt is neglected, the transverse displacements are considerably smaller than the distance between the idlers, and the belt’s length change due to transverse deformation is negligible compared to the belt’s initial length. In essence, Lodewijks’ model can be considered a hybrid of string and beam models, as it incorporates some of the assumptions of both models.

Harrison’s conveyor belt vibrations model^51^ is a complex mathematical model used to calculate the frequency of transverse vibrations in conveyor belts. The model is based on the mechanics of the plate and considers the stiffness of the belt and the boundary conditions. Some solutions of the model also include a belt stretching force. It involves developing a flat belt and a belt supported on two or three rollers to determine the vibrations. The model assumes that the belt is fixed on the bend line between the idlers and recommends the use of software to determine the frequency of vibrations^52^. Harrison’s model shows greater accuracy than the string model, but it is complicated and requires many assumptions to be made for the calculations.

In terms of designing belt conveyors, the string model which includes Lodewijks beam elements, is used in computer-aided calculations and is favored by some design companies for conveyor route design. Empirical evidence suggests that under certain operational conditions, the inaccuracies observed in the predictions generated by the FEM model may be attributed to the absence of belt rigidity in the model. Harrison’s vibration model, on the other hand, is known for its high degree of complexity and is typically used for more detailed calculations where recalculating auxiliary coefficients and selecting determinants defining the types of vibrations is necessary and justified. Overall, both models have their uses and are employed in different scenarios based on their strengths and limitations. Accordingly, efforts have been directed towards formulating a novel model that accounts for all the pertinent features of conveyor belts, while simultaneously possessing sufficient simplicity to be utilized in fundamental belt conveyor design. One of the fundamental assumptions underlying the development of a new model was the simplification of calculations and the elimination of subjective parameters. This was motivated by the fact that several existing models involve the incorporation of stiffness coefficients^53,54^ of both the conveyor and the belt into their equations.

## Flexural rigidity of belt

**Figure 1:**
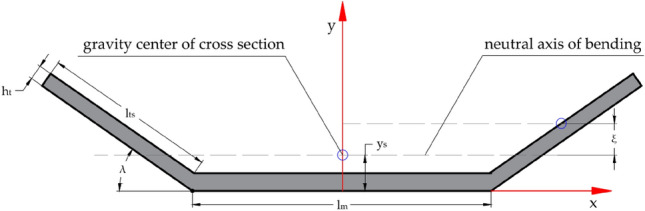
The cross-section of the belt at the symmetrical three-roller trough.

Flexural rigidity is a parameter that determines the line of bending of the conveyor belt on the idler supports. Proper alignment of the unloaded belt is a prerequisite for the correct operation of the conveyor. If the belt is not properly aligned, it may slip or become damaged, leading to downtime and increased maintenances. Usually, the transverse rigidity is determined analytically based on the geometry of the cross-sectional profile and the material parameters of the belt. It can be expressed as the product of the moment of inertia of the cross-section *I* and the modulus of elasticity *E*. The transverse rigidity of the belt depends on the way the belt is positioned on the idlers. The number and arrangement of idlers on the carrying and return sides of the belt loop are different, which is why the rigidity is also variable. The most general case of guiding the belt on idler supports is the symmetrical arrangement, three-roller support, used on the carrying side of the belt loop, which was considered in the article (Fig. 1). Additional parameters that affect changes in transverse rigidity include the trough angle, belt thickness and width, and idler length. In the calculations of the flexural rigidity of the cross-section of the belt, it is assumed that the neutral bending axis is a horizontal line passing through the center of gravity of the cross-sectional profile of the belt.

In the symmetrical system of the three-roller basin, the location of the center of gravity of the cross-section is determined by the coordinate:

1 $$\begin{aligned} y_s=\frac{l_{ts}^2 \cdot \sin \lambda }{l_m+2 \cdot l_{ts}}=\frac{l_{ts}^2}{B} \cdot \sin \lambda \end{aligned}$$

The centre of gravity of the lateral section of the trough $l_{ts}^2$ is shifted vertically from the neutral axis of bending by the size of:

2 $$\begin{aligned} \xi =\frac{1}{2} \cdot l_{ts}^2 \cdot \sin \lambda - y_s=l_{ts} \cdot \left( \frac{1}{2} - \frac{l_{ts}^2}{B}\right) \cdot \sin \lambda \end{aligned}$$

To determine the moment of inertia, the calculations must be divided into two parts. In the first stage, the moment of inertia of the section of the trough inclined relative to the neutral axis is determined:

3 $$\begin{aligned} I_s=\frac{h_t \cdot l_{t s}^3}{12} \cdot \sin ^2 \lambda + h_t \cdot l_{t s} \cdot \xi ^2 = h_t \cdot l_{t s}^3 \cdot \sin ^2 \lambda \cdot \left[ \frac{1}{3}-\frac{l_{t s}}{B}+\left( \frac{l_{t s}}{B}\right) ^2\right] \end{aligned}$$

In the second stage, the moment of inertia of the central part can be determined using the following formula:

4 $$\begin{aligned} I_m=h_t \cdot l_m \cdot l_{t s}^2 \cdot \left( \frac{l_{t s}}{B}\right) ^2 \cdot \sin ^2 \lambda \end{aligned}$$

In the final calculation, the moment of inertia of the cross-section of the troughed belt is equal to: $I=I_m + 2 \cdot I_s$.

## The MCB model

### Equation of motion

**Figure 2:**
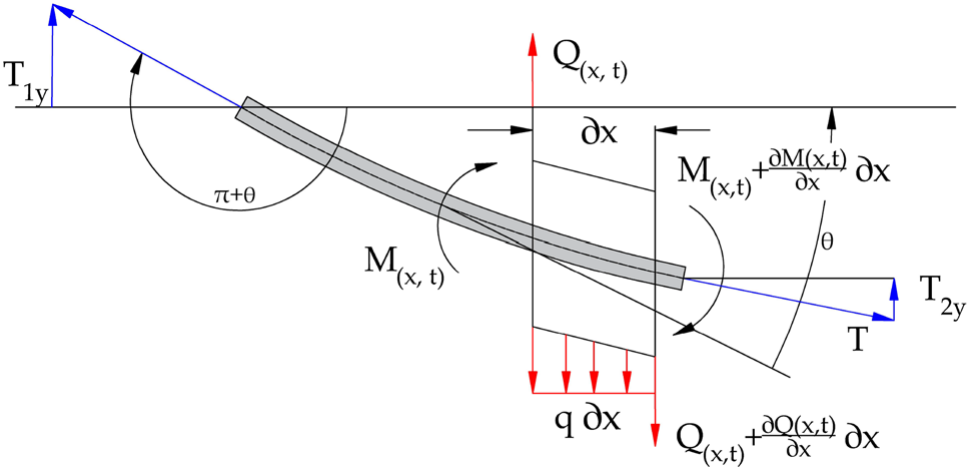
The section of the belt cross-section of length *dx*.

In the analyzed model, the conveyor belt was treated as a homogeneous beam with a constant cross-section. Double-sided support of the belt was used. The conveyor belt as a beam is simply supported, from left side ($x=0$), to the right ($x=l$). The belt performs bending vibrations and the cross-section displacement is defined as $y=y(x,t)$. Figure 2 shows a section of the belt cross-section of length *dx*, loaded with acting forces, which allowed to define the equation of motion.

The equation of motion for the analyzed cross-section in the displacement direction is equal:

5 $$\begin{aligned} d m a=-Q(x, t)+Q(x, t)+d Q(x, t)-T \frac{\partial y(x, t)}{\partial x}+T \frac{\partial y(x, t)}{\partial x}+T \frac{\partial ^{2} y(x, t)}{\partial x^{2}} \end{aligned}$$

The equation (5) makes sense for assuming a linear-elastic material and ignoring the circular motion of the section. In real conditions, conveyor belts are composites with a nonlinear Young’s modulus, with additional material damping^55,56^. Due to the fact that the estimation of the frequency of transverse vibrations is usually carried out for a limited range of belt stresses, a segmental linearization of the longitudinal elasticity characteristic can be performed. Considering these assumptions, the mass of the vibrating element in Equation (5) can be defined as $d m=\rho S d x$, included in the equation (5) acceleration of the *dx* element is $a=\frac{\partial ^{2} y(x, t)}{\partial t^{2}}$, next the transverse force is equal to $Q(x, t)=\frac{\partial M(x, t)}{\partial x}$, and the bending moment can be related to the radius of curvature resulting from the cyclic displacements of the beam $\frac{M}{E I}=\frac{1}{r}$, we we can define the condition that small deflections determine small angles between the horizontal axis and the direction of force along the beam axis $\frac{1}{r(x, t)}=-\frac{\partial ^{2} y(x, t)}{\partial x^{2}}$. Taking into account all the presented assumptions, we can write that the shear force (transverse) is:

6 $$\begin{aligned} d Q(x, t)=\frac{\partial Q(x, t)}{\partial x} d x=-\frac{\partial }{\partial x^{2}}\left[ E I \frac{\partial ^{2} y(x, t)}{\partial x^{2}}\right] d x \end{aligned}$$

All presented relations can now be substituted into the original equation of motion (5), and the ordering of the variables and the degree of derivatives gives the following solution:

7 $$\begin{aligned} E I \frac{\partial ^{4} y(x, t)}{\partial x^{4}}-T \frac{\partial ^{2} y(x, t)}{\partial x^{2}}+\rho S \frac{\partial ^{2} y(x, t)}{\partial t^{2}}=0 \end{aligned}$$

The first part of the equation (7) is the increment of the shear force in the beam along the length of *dx*. The second part describes the force with which the tensioned belt resists the movement of the element. The third part is the elementary force of inertia. The standard solution of the equation of motion (7), which does not take into account the axial stress, leads to the following formula for the frequency of bending vibrations of the beam:

8 $$\begin{aligned} f=\frac{\pi }{2 l^{2}} \sqrt{\frac{E I}{\rho S}} \end{aligned}$$

### Prestressing concept

The solution presented in the previous subsection completely ignores the influence of longitudinal forces in the belt and its speed of movement. The method of taking into account the axial forces assumes a two-sided action of the compressive force^57^. The force acting along the axis of the deflected beam (curve) is equal to:

9 $$\begin{aligned} T_{y}=T_{1 y}+T_{2 y}=T \sin (\pi +\theta )+T \sin \theta _{1} \end{aligned}$$

Due to the fact that the transverse deflections of the belt are relatively small, it can be assumed that the angles formed between the horizontal plane are also very small and thus their influence is neglected. For such assumptions, the following conditions can be written: $\sin \theta \cong \theta$ and $\sin \theta _{1}=\theta _{1}=\theta (x, t)+\frac{\partial \theta (x, t)}{\partial x} d x$. The equation (9) therefore takes the following form:

10 $$\begin{aligned} T_{y}=T \theta +T\left( \theta +\frac{\partial \theta }{\partial x} d x\right) =T \frac{\partial ^{2} y(x, t)}{\partial x^{2}} \end{aligned}$$

For this reason (10) the equation of motion (7) is also appropriate for a beam under double compression. The normal form of vibration as a function of displacement and time can be written as follows^58^: $y=\eta (x) f(t)=\eta \sin \alpha t$. By inserting into the equation of motion (7), after performing a few transformations it is possible to derive the equation:

11 $$\begin{aligned} \frac{\partial ^{4} \eta (x, t)}{\partial \xi ^{4}}-\gamma _{1}^{2} \frac{\partial ^{2} \eta (x, t)}{\partial \xi ^{2}}-\beta ^{4} \eta (x, t)=0 \end{aligned}$$

In the equation (11) $\beta =l \root 4 \of {\frac{\rho S \alpha ^{2}}{E I}}$, $\gamma _{1}=l \sqrt{\frac{T}{E I}}$, and $\xi =\frac{x}{l}$. To solve this equation, we first assume a solution of the form $\eta (x) = A\sin (\omega x)+B\cos (\omega x)+C\sinh (\omega x)+D\cosh (\omega x)$, where *A*, *B*, *C*,  and *D* are constants to be determined and $\omega$ is the natural frequency of the system. After solving the equation, the frequency of bending vibrations of an axially prestressed beam under double compression is obtained as:

12 $$\begin{aligned} f=\frac{\pi }{2 l^{2}} \sqrt{\frac{E I}{B m}} \cdot \sqrt{1+\frac{T l^{2}}{\pi ^{2} E I}} \end{aligned}$$

In the case of conveyor belts, the use of material density and cross-sectional area is not practical. A better solution is to replace the term $\rho S$ with an expression *Bm*.

### Motion concept

**Figure 3:**
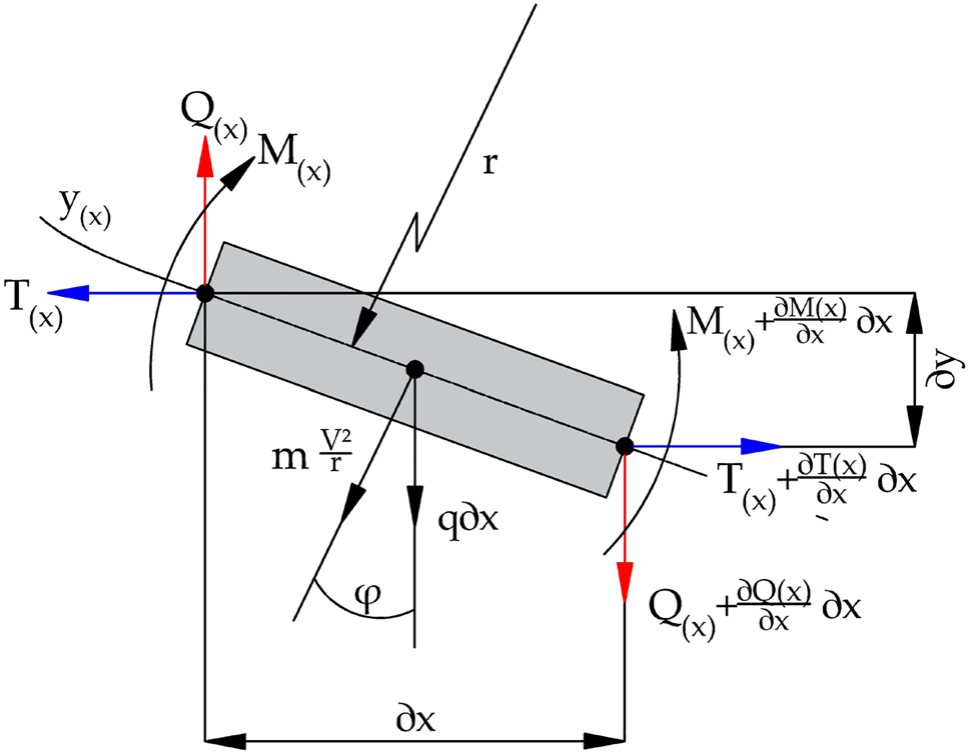
The load on the separated belt element with the length *dx*.

Taking into account the variable stresses is a procedure that allows to distinguish the specific features of the string models, while maintaining the material properties of the belt. The Fig. 3 shows the load distribution for a short section of the belt length^59^.

The equation follows from the balance of forces in the vertical direction acting on the separated belt element:

13 $$\begin{aligned} -d Q(x)=m g d x+m \frac{v^{2}}{r} \cos \varphi d x \end{aligned}$$

As in the case of the equation (10), an assumption of small, negligible deflections can be made: $$\cos \varphi \cong 1$$ and $$r=-\frac{1}{\frac{\partial ^{2} y(x)}{\partial x^{2}}}$$. In this case, equation (13) can take form: $$\frac{\partial Q(x)}{\partial x}=m v^{2}-m g$$. The transverse forces in the belt depend on the mass of the analyzed section and the speed. On this basis, it is possible to derive a parameter called the equivalent tensile force in the belt: $$ T_{0}=T-m v^{2}$$. The equivalent belt tensile force is reduced in relation to the belt force by the normal inertia force (centrifugal force) acting on the transported material and the belt. By introducing (12) into the equations we obtain the final formula for the frequency of transverse vibrations of a movable, axially loaded conveyor belt:

14 $$\begin{aligned} f=\frac{\pi }{2 l^{2}} \sqrt{\frac{E I}{B m}} \cdot \sqrt{1+\frac{T_{0} l^{2}}{\pi ^{2} E I}}=\frac{\pi }{2 l^{2}} \sqrt{\frac{E I}{B m}} \cdot \sqrt{1+\frac{\left( T-B m v^{2}\right) l^{2}}{\pi ^{2} E I}} \end{aligned}$$

## Model sensitivity

**Figure 4:**
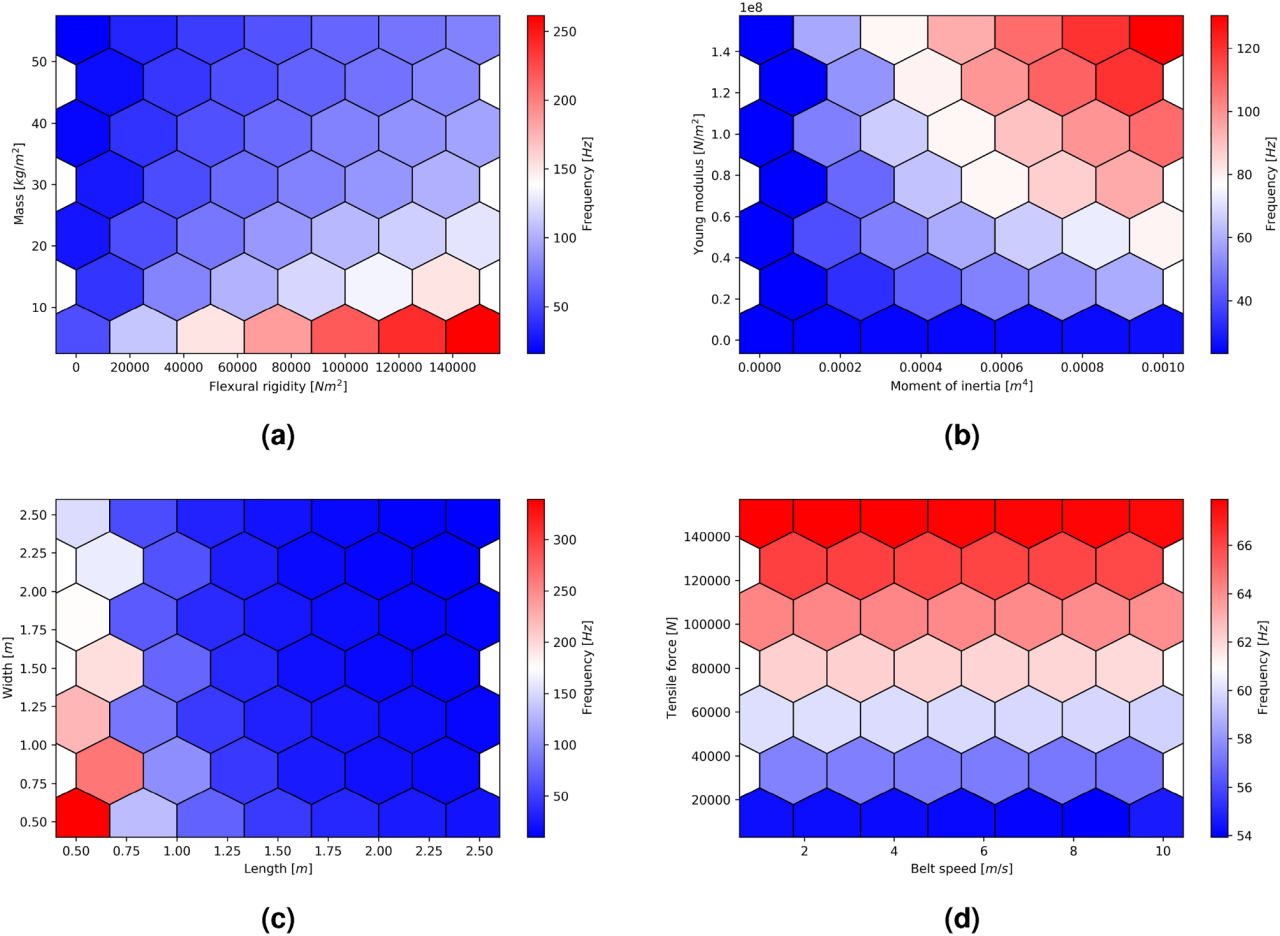
Sensitivity analysis of the MCB model: (**a**), (**b**) for changing material parameters, (**c**) for changing construction parameters, (**d**) for changing operating parameters.

The parameters that affect conveyor belt transverse vibration can be divided into three groups. The first group includes material parameters of the belt, such as flexural rigidity and mass. The second group comprises geometric or structural parameters, such as belt width and support spacing. Finally, the third group consists of technological and operational parameters, such as belt speed and tensioning force, which can affect the belt’s behavior during normal operation. The sensitivity analysis results reveal the parameters that exert the most significant influence on the frequency of transverse vibrations of the belt, highlighting their crucial role in the initial stages of belt conveyor design By systematically varying these parameters and observing their impact on the belt’s vibration behavior, sensitivity analysis can help optimize the design and operation of conveyor belt systems to reduce vibration and improve performance. Figure 4 shows the results of the model sensitivity analysis for three groups of input parameters. The range of parameters was selected based on the actual working conditions of the conveyors^60^.

The results indicate that the transverse flexural rigidity of the belt, one of the material parameters included in the model, has the greatest impact on the frequency outcome (Fig. 4a). In comparison to the effect of flexural rigidity, the impact of belt mass on the resulting frequency outcomes is insignificant. However, it is important to note that there is a considerable variation in the examined parameter ranges, which are derived from the conventional operating conditions of the conveyor. The mass of the belt is typically linked to its nominal strength, which in turn influences the elastic modulus. The flexural rigidity of a belt is dependent on the cross-sectional area’s moment of inertia and the modulus of elasticity. The findings indicate that, among the examined parameters, the impact of the cross-sectional geometry has the most substantial effect on the vibration frequency (Fig. 4b). This effect is non-linear, highlighting a marked increase in the belt’s vibration frequency at greater trough angles.

Moving on to construction parameters (Fig. 4c), the belt width was found to be the most decisive factor in changing the transverse vibration frequency. Furthermore, the idler spacing was identified as another design parameter that has a noticeable impact on the vibration frequency. Although the effect is smaller compared to belt width, it should not be overlooked, and designers must carefully consider this parameter when designing conveyor systems. Notably distinct alterations are noticeable for short support spacings.

Finally, among the operating parameters (Fig. 4d), the belt tensile force was identified as the main factor that affects the transverse vibration frequency. In contrast, the effect of speed on the vibration frequency was found to be minimal. Based on these findings, it can be confidently affirmed that selecting the appropriate belt tensioning force and managing belt tension during steady states are crucial for achieving optimal vibration characteristics of the conveyor.

**Figure 5:**
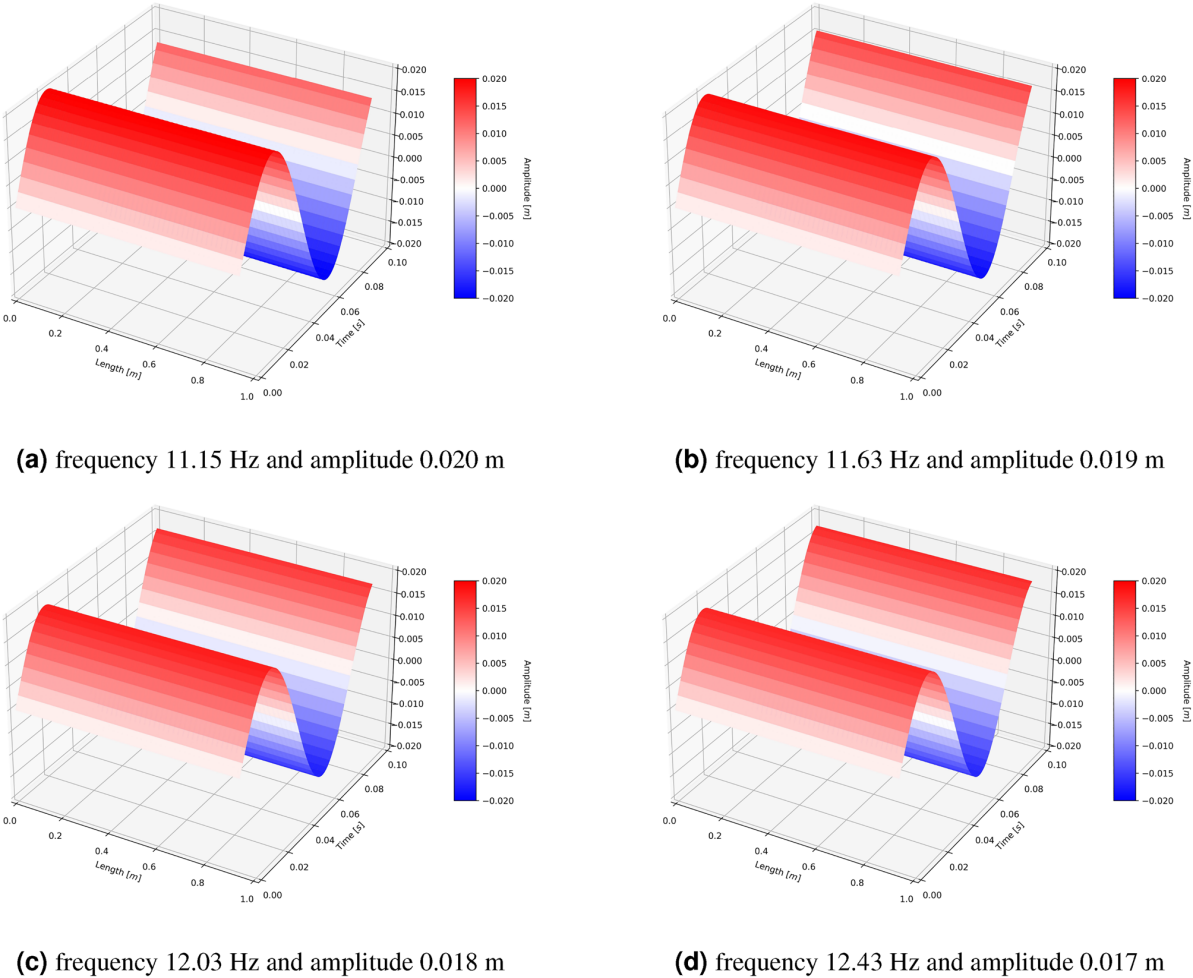
The MCB model solutions in the time domain on the length between the supports for standard conveyor belt trough angles: (**a**) 30°, (**b**) 35°, (**c**) 40°, (**d**) 45°.

The outcomes of the sensitivity analysis of the model provide clear evidence of the significant impact of the belt cross-sectional geometry on the transverse vibrations of the belt. Thus, when designing the conveyor capacity, the trough angle selection should also be considered during the stage of safeguarding the conveyor against resonance. The value of the trough angle ought to be incorporated into the calculations of the tensile resonance force. To demonstrate the effect of the trough angle on the vibration characteristics, Fig. 5 presents the model’s solution for a brief time interval using standard trough angles, and assuming a sinusoidal course with a standard function of amplitude. The belt parameters, which were utilized in laboratory tests to validate the model, were employed as input data to solve the equations.

## Materials and methods used in validation

### Equipment

**Figure 6:**
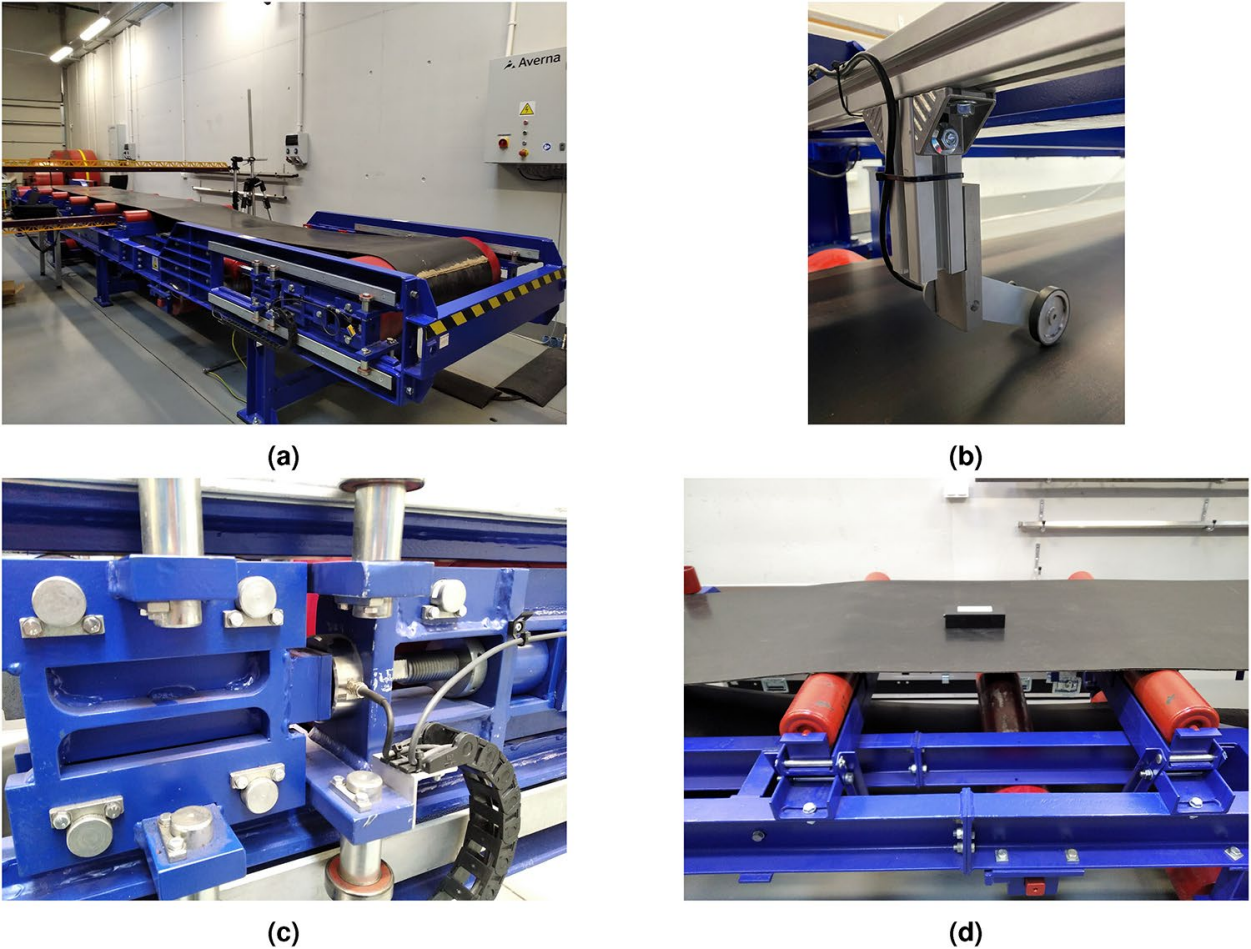
Equipment used to MCB model validation: (**a**) test rig, (**b**) encoder for belt speed measurement, (**c**) load cell for measuring the tensile force of the belt, (**d**) measuring device on a moving conveyor bel.

The proposed model and theoretical results of the sensitivity analysis were verified through laboratory experimentation on a conveyor (Fig. 6a) with an approximate length of 8 meters. The conveyor consists of five idler sets, with each set having three flat-tubed steel rollers with a diameter of 133 mm and a length of 300 mm, that can be inclined in five different positions. The average distance between each set is 0.93 meters. The rig is capable of smoothly adjusting the belt speed up to 5.3 m/s. During the experimentation, a linear speed encoder with a wheel (Fig. 6b) was used to measure the belt’s speed, while a hydraulic tension system applied a constant tensile force to the belt. The tensile force was measured by strain gauge force sensors located on the return pulley (Fig. 6c). The study utilized mobile measurement devices that traverse the entire conveyor route during operation, as depicted in Fig. 6d. These devices consist of a vibration sensor enclosed in a sealed casing that is mounted to the conveyor belt. A microcontroller performs the acquisition of measurement data, initial data processing, and data storage on an SD card. The system operates on independent battery power and features a free fall detection mechanism to monitor for unintended contact losses between the device and the belt. The device’s weight is sufficiently low so as not to cause significant point changes in the weight of the belt. Sampling is conducted at a rate of 2,5 kHz.

The experiments were conducted on a textile belt with EP250 specifications, with a length of approximately 17 meters. A Table  1 was prepared summarizing all the relevant parameters required for the computational model.

**Table 1:** Properties of the EP250 belt.

Parameter	Value
Width	$0.8 \mathrm {~m}$
Thickness	$5.69 \mathrm {~mm}$
Amount of plies	2
Mass	$7.19 \mathrm {~kg} / \textrm{m}^{2}$
Young’s module	$3.75 \cdot 10^{6} \mathrm {~N} / \textrm{m}^{2}$
Nominal tensile strength	$250 \mathrm {~kN} / \textrm{m}$

### Methods

The study involved capturing vibration time series data using a device that was placed on a conveyor belt under different trough angles. By adjusting the trough angle, the stiffness of the belt cross-section was modified. Data was collected for five different trough angles: 0$^{\circ }$, 27$^{\circ }$, 31$^{\circ }$, 38$^{\circ }$, and 41$^{\circ }$, with the flat belt run designated as angle 0$^{\circ }$. Although the flat belt run is not commonly used for the upper belt during bulk material transportation (except in specific transition sections), it serves as an interesting reference point for model validation since it leads to a collapse of the applied geometrical relations. The measurement device traversed the entire route from the return pulley to the drive pulley, capturing the vibration signal of the entire conveyor. However, only short time segments located between the idler sets were selected for processing. The exact distances between the supports in this location were known. Short time periods were subjected to processing (filtration and spectral analysis) in order to extract the characteristic frequencies of the components. Filtering is necessary to separate interference in the extremely low frequency range. To accomplish this, a high-pass filter was utilized. The final course of the transverse vibration frequency as a function of the trough angle was determined and compared with the model proposed in the article. Full statistics of measured frequencies and amplitudes of vibrations for all series and measurement points are presented. The average forecast error of the proposed model and the popular models used so far were determined. The discussion section formulates conclusions based on the results obtained. The tests were carried out for a constant belt tension force of $2.0~\textrm{kN}$ and speed $1.3~\mathrm {m/s}$. Under the specific operating conditions of the conveyor, an estimated measurement error of $\pm ~0.1~\mathrm {m/s}$ was determined for belt speed, and $\pm ~0.1~\textrm{kN}$ for belt force.

## Results of model validation

**Figure 7:**
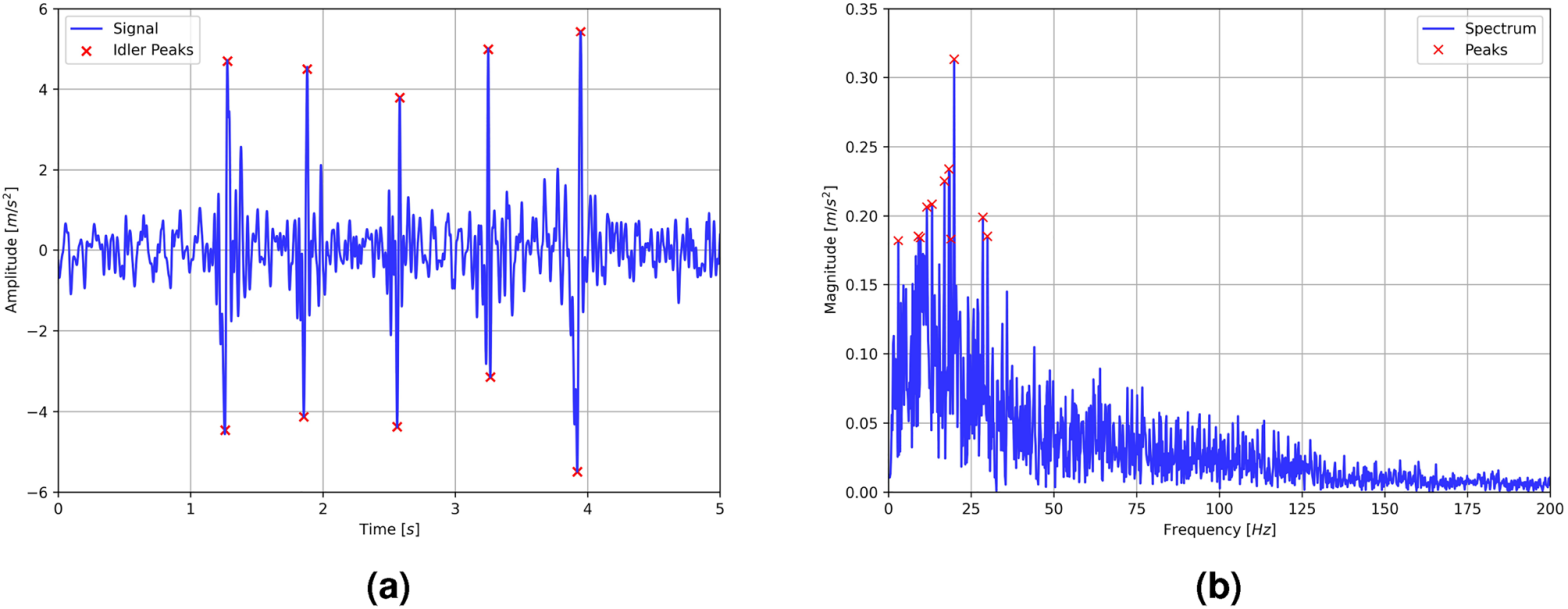
The recorded signal of transverse vibrations from the entire conveyor: (**a**) signal over time with the identified peaks corresponding to the idler supports and the analysis areas, (**b**) frequency spectrum of a signal with the most significant frequencies.

Figure 7 shows an exemplary full time signal (Fig. 7a) and spectrum (Fig. 7b) of the recorded vibration signal from the entire conveyor. The detected peaks are attributed to direct interaction between the measuring device and the idler, as the device passes through the idler. The recorded frequencies of various phenomena along the conveyor path, including belt vibrations in different directions, interaction between the belt and idler, and transmission of vibrations from other systems, fall within the frequency range of 125 Hz, with intensification up to 50 Hz. The observed peaks in frequency resulting from excitation generated by the rotating rollers are within the range of 10 to 20 Hz.

**Figure 8:**
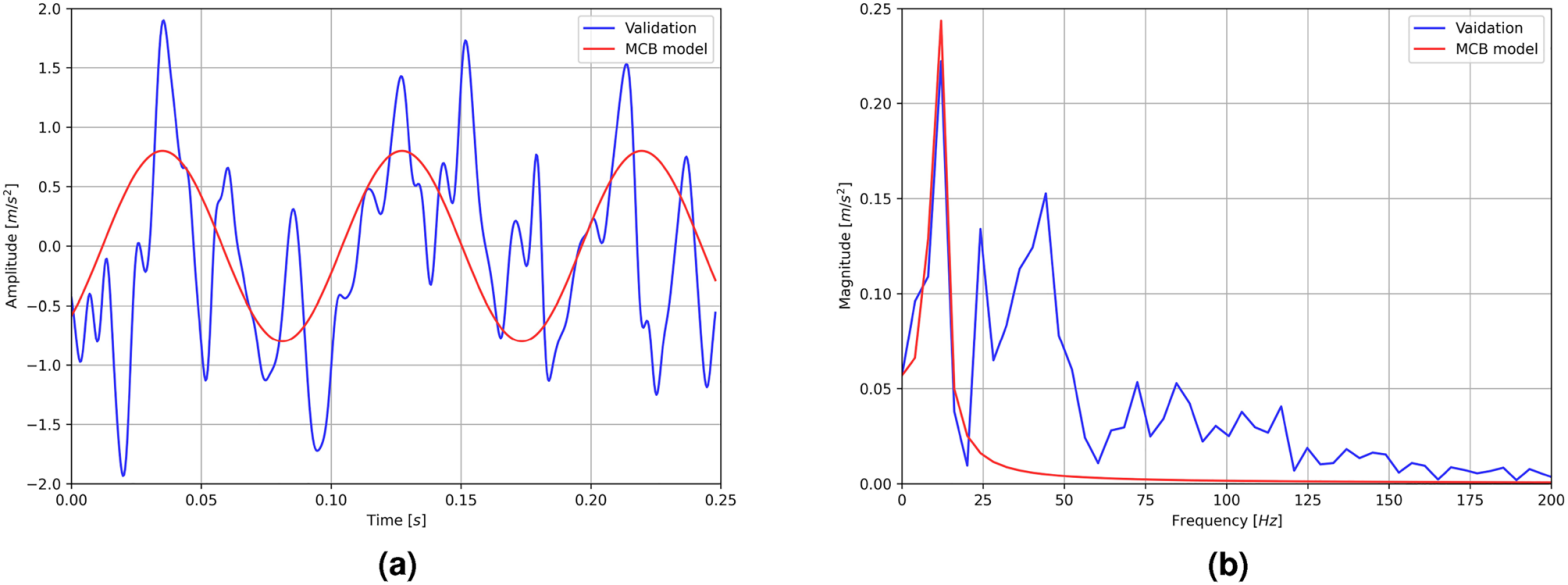
The recorded transverse vibration signal for a particular section that is located between the supports of the idler: (**a**) time signal, (**b**) signal spectrum.

The following Fig. 8 depicts a sample recorded signal for the segment of the conveyor belt located between the idler.

## Discussion

**Figure 9:**
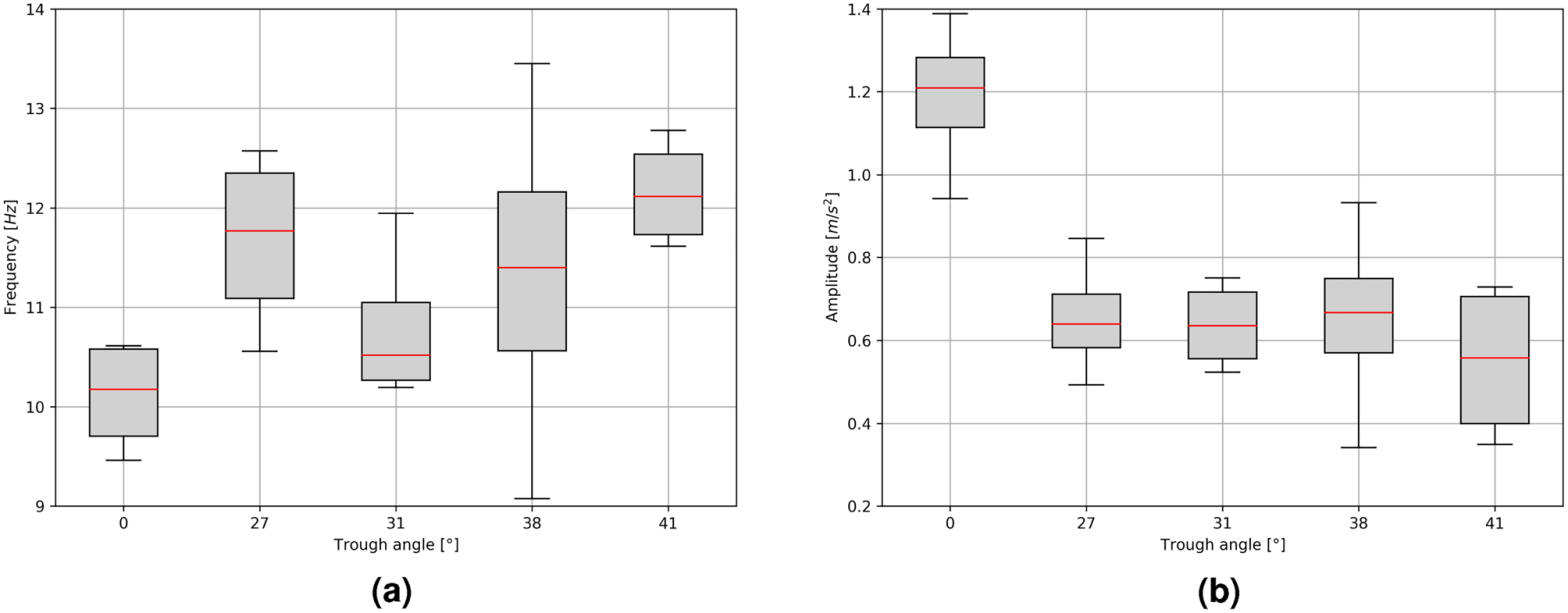
Measurement results for each analyzed trough angle: (**a**) frequency, (**b**) ampitude.

By filtering out short waveforms and removing disturbances recorded directly from the belt, it becomes possible to identify trends in the amplitudes of transverse vibrations between the supports. The frequencies of these vibrations for each trough angle are determined and presented in a box plot in Fig. 9a, along with full statistics. In the Fig. 9b, the statistics of the recorded average vibration amplitudes are presented in a similar way as in the case of the transverse vibration frequency. The recorded vibration amplitudes pertain to the entire structure of the conveyor and not just to small sections of the belt between the idlers.

The measured values of the frequency of the transverse vibrations of the belt between the idler supports, for these specific settings of the conveyor, were in the approximate ranges from 10 Hz to 12 Hz. With the increase in the trough angle (and thus the exponen- tial increase in the rigidity of the belt cross-section), the measured vibration frequencies slightly increased. The amplitude of the belt vibrations decreased with the increase of the trough angle. This is due to the increase in the stiffness of the belt cross-section and the lower tendency of the belt to lateral and horizontal movements. In the study of the tests carried out for the unloaded belt, it can be concluded that large trough angles better stabilize the belt.

**Figure 10:**
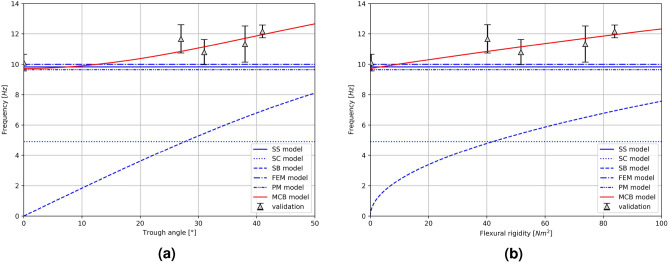
Measured vibration frequencies and comparison with other models: (**a**) as a function of the trough angle, (**b**) as a function of rigidity.

The measured transverse vibration frequencies were averaged and compared on a common plot (Fig.  10) with the forecasts of the MCB model, the standard string model (SS model)^61^, the string model used so far for the design of conveyors (SC model)^59^, the standard model of the axially unloaded beam (SB model)^57^, the string model with beam elements (FEM model)^50^ and the vibrating plate model (PM model)^51^. The results are shown as a function of the trough angle (Fig.  10a) and as a function of rigidity (Fig.  10b).

The study found that the MCB model has a mean absolute error of 0.79 Hz in predicting the vibration frequency of conveyors, with respect to the expected values based on validation data. The study revealed that the SS, FEM, and PM models exhibited the highest accuracy among the other models evaluated, as they consistently predicted a constant vibration frequency around 10 Hz. The level of proximity to the validation measurements was sufficiently close that the suitability of these models in conveyor design cannot be dismissed. The study found that for the flat belt, the FEM model exhibited superior accuracy compared to the MCB model. However, it is not possible to conclusively state whether the FEM model provides better prediction accuracy for small trough angles, due to the absence of additional validation points and the unique characteristics of the flat belt operation. Within the range of typical trough angles used in conveyor design, the other models evaluated in the study lacked sensitivity to changes in the flexural rigidity of the belt cross-section. On the other hand, the MCB model exhibited a high degree of accuracy, closely tracking the validation data and providing the most precise predictions among the design tools tested. The SS and SB models were found to consistently predict vibration frequencies approximately 1.5 to 2 times lower than the actual validation results^62^.

## Conclusion

In conclusion, the vibration analyses of the conveyor belt have revealed that existing calculation models often neglect the real influence of belt stiffness on the vibration frequency. The theoretical analyses suggest that the conveyor belt behaves like a beam with bending stiffness. The geometry of the belt, particularly the presence of a trough, increases the inertia of the cross-section, resulting in higher bending stiffness compared to a flat belt configuration. The stiffness of the belt cross-section is directly correlated with the angle of the trough. Laboratory tests have provided evidence that even for a belt of low strength, there is an observable increase in the stiffness of the cross-section bending as the trough angle increases.

The model was found to be directly applicable to the conveyor system under discussion. Furthermore, changing the rigidity of the cross-section, achieved by modifying the trough angle, affected the frequency and amplitude of transverse vibrations of the unloaded belt, and the proposed model provided the smallest error while maintaining the correct changes according to the rigidity of the cross-section.

Due to the model’s small number of determinable parameters and the lack of dimensionless coefficients regulating the stiffness of the conveyor-belt system, it can be effectively used in the engineering design stage to prevent resonance and is a more precise alternative to commonly used solutions. The research conducted using the developed model on a real object in the rock aggregates mine has yielded qualitative results, which have been presented in a publication.

## List of Symbols

*I*: Moment of inertia of the belt cross-section
*E*: Young’s modulus of elasticity
$I_s$: Moment of inertia of the inclined part of the cross-section of the belt
$$h_t$$: Belt thickness
$l_{ts}$: Length of the inclined side roller
$\lambda$: Angle of inclination of the belt trough
*B*: Belt width
$I_m$: Moment of inertia of the central part of the cross-section of the belt
$l_m$: Length of the center roller
*m*: Mass
*a*: Acceleration
*Q*: Shear (transverse) force
*T*: Axial force
$$ \rho $$: Density
*S*: Cross-sectional area
*M*: Bending moment
$\theta _{1 y}, \theta _{2 y}$: The angles between the horizontal axis and the direction of force*T*
*g*: Standard acceleration due to gravity equilibrium position
*v*: Belt speed
$\varphi$: The angle between the vertical axis and the direction of the centrifugal force
$T_{0}$: Equivalent belt tensile force
*EI*: Flexural rigidity
*r*: Radius of curvature
*l*: Support distance (spacing)
$\theta$: Angle of inclination from equilibrium
$T_{1 y}, T_{2 y}$: Vertical components of the *T* force
$\xi$: Distance between center of gravity of lateral section of belt trough and the center of gravity of belt trough cross section
$y_s$: Location of gravity center relative to the vertical axis *y* of the coordinate system

## References

[1] Yan, C. & He, X. Model and dynamic simulation of belt conveyor. *Proc. 2010 International Conference on Intelligent System Design and Engineering Application*, vol. 1, 949–951, 10.1109/ISDEA.2010.331 (IEEE, 2010).

[2] Harrison A (1992). Modern design of belt conveyors in the context of stability boundaries and chaos. Philos. Trans. R. Soc. London Ser. A Phys. Eng. Sci..

[3] Rana S, Nagayama T, Hisazumi K, Tominaga T (2019). Damage identification of a belt conveyor support structure based on cross-sectional vibration characteristics. Struct. Control. Health Monit..

[4] Zimroz R, Król R (2009). Failure analysis of belt conveyor systems for condition monitoring purposes. Min. Sci..

[5] Homišin J, Grega R, Kaššay P, Fedorko G, Molnár V (2019). Removal of systematic failure of belt conveyor drive by reducing vibrations. Eng. Fail. Anal..

[6] Chen L-Q (2005). Analysis and control of transverse vibrations of axially moving strings. Appl. Mech. Rev..

[7] Hu, Y., Wang, L., Wang, X., Qian, X. & Yan, Y. Simultaneous measurement of conveyor belt speed and vibration using an electrostatic sensor array. *Proc. 2015 IEEE International Instrumentation and Measurement Technology Conference (I2MTC)*, 757–761, 10.1109/I2MTC.2015.7151363 (IEEE, 2015).

[8] Hou Y-F, Meng Q-R (2008). Dynamic characteristics of conveyor belts. J. China Univ. Min. Technol..

[9] He D, Pang Y, Lodewijks G (2016). Belt conveyor dynamics in transient operation for speed control. Int. J. Civ. Environ. Struct. Constr. Archit. Eng..

[10] Li J, Pang X (2018). Belt conveyor dynamic characteristics and influential factors. Shock Vib..

[11] Harrison A (1985). Reducing dynamic loads in belts powered by three wound-rotor motors. Bulk Sol. Handl..

[12] Bauomy H, El-Sayed A (2018). Vibration performance of a vertical conveyor system under two simultaneous resonances. Arch. Appl. Mech..

[13] Brown, S. Conveyor noise specification and control. * Proc. Conference paper 2004 Annual Conference of the Australian Acoustical Society 3e5 November*, 269e76 (2004).

[14] Harrison, A. & Roberts, A. Technical requirements for operating conveyor belts at high speed. *Bulk Solids Handl. (Germany)* **4** (1984).

[15] Ganapathy S, Parameswaran M (1987). Effect of material loading on the starting and transition over resonance of a vibratory conveyor. Mech. Mach. Theor..

[16] Roos WA, Heyns PS (2021). In-belt vibration monitoring of conveyor belt idler bearings by using wavelet package decomposition and artificial intelligence. Int. J. Min. Miner. Eng..

[17] Guo YC, Cheng G, Hu K, Wang ZF (2012). Research on the idler spacing of belt conveyor. Appl. Mech. Mater..

[18] Waters, A. & Mikka, R. Segregation of fines in lump ore due to vibration on a conveyor belt. * Proc. Third International Conference on Bulk Materials, Storage, Handling and Transportation: Preprints of Papers: Preprints of Papers*, 89–93 (Institution of Engineers, Australia Barton, ACT, 1989).

[19] Park S-T, Yang B-S (2010). An implementation of risk-based inspection for elevator maintenance. J. Mech. Sci. Technol..

[20] Rodríguez JR (2005). Resonances in a high-power active-front-end rectifier system. IEEE Trans. Ind. Electron..

[21] Golka K, Bolliger G, Vasili C (2007). Belt Conveyors: Principles for Calculation and Design.

[22] Ding H, Tang Y-Q, Chen L-Q (2017). Frequencies of transverse vibration of an axially moving viscoelastic beam. J. Vib. Control.

[23] Ding H, Zu JW (2013). Effect of one-way clutch on the nonlinear vibration of belt-drive systems with a continuous belt model. J. Sound Vib..

[24] Hu Y, Yan Y, Wang L, Qian X (2016). Non-contact vibration monitoring of power transmission belts through electrostatic sensing. IEEE Sens. J..

[25] Zaharov AY, Erofeeva NV (2015). Vibration of the belt and workflows of the conveyor. Vestn. Kuzbass State Tech. Univ..

[26] Bartelmus, W. & Sawicki, W. Progress in quality assessment of conveyor idlers. *Proc. of the 16th IMEKO World Congress* (Vienna, Austria, 2000).

[27] Reicks AV (2008). Belt conveyor idler roll behaviors. Bulk Mat. Handl. Conveyor Belt.

[28] Yang G (2014). Dynamics analysis and modeling of rubber belt in large mine belt conveyors. Sens. Transducers.

[29] Beikmann, R.S. *Static and dynamic behavior of serpentine belt drive systems: Theory and experiment* (University of Michigan, 1992).

[30] Ulsoy AG, Whitesell JE, Hooven MD (1985). Design of belt-tensioner systems for dynamic stability. J. Vib. Acoust. Stress. Reliab. Des..

[31] Barker CR, Oliver LR, Breig WF (1991). Dynamic analysis of belt drive tension forces during rapid engine acceleration. SAE Trans..

[32] Beikmann RS, Perkins NC, Ulsoy AG (1996). Free vibration of serpentine belt drive systems. J. Vib. Acoust..

[33] Callegari M, Cannella F, Ferri G (2003). Multi-body modelling of timing belt dynamics. Proc. Inst. Mech. Eng. Part K J. Multi-body Dyn..

[34] Leamy MJ, Wasfy TM (2002). Transient and steady-state dynamic finite element modeling of belt-drives. J. Dyn. Sys. Meas. Control.

[35] Mote C, Wu W (1985). Vibration coupling in continuous belt and band systems. J. Sound Vib..

[36] Mockensturm EM, Guo J (2005). Nonlinear vibration of parametrically excited, viscoelastic, axially moving strings. J. Appl. Mech..

[37] Abrate S (1992). Vibrations of belts and belt drives. Mech. Mach. Theor..

[38] Eliseev V, Vetyukov Y (2012). Effects of deformation in the dynamics of belt drive. Acta Mech..

[39] Límaco J, Clark H, Medeiros L (2008). Vibrations of elastic string with nonhomogeneous material. J. Math. Anal. Appl..

[40] Suweken G, Van Horssen W (2003). On the transversal vibrations of a conveyor belt with a low and time-varying velocity. Part I the string-like case. J. Sound Vib..

[41] Harrison, A. Future design of belt conveyors using dynamic analysis. *Bulk Solids Handling* **7** (Germany, Federal Republic, 1987).

[42] Zhu F, Parker RG (2008). Influence of tensioner dry friction on the vibration of belt drives with belt bending stiffness. J. Vib. Acoust..

[43] Andrianov IV, Van Horssen WT (2008). On the transversal vibrations of a conveyor belt: Applicability of simplified models. J. Sound Vib..

[44] Pan Y, Liu X, Shan Y, Chen GS (2017). Complex modal analysis of serpentine belt drives based on beam coupling model. Mech. Mach. Theor..

[45] Zamiralova ME, Lodewijks G (2017). Review of the troughability test ISO 703 for quantifying a uniform transverse bending stiffness for conveyor belts. Arch. Civ. Mech. Eng..

[46] Scurtu PR, Clark M, Zu JW (2012). Coupled longitudinal and transverse vibration of automotive belts under longitudinal excitations using analog equation method. J. Vib. Control.

[47] Zheng Q, Xu M, Chu K, Pan R, Yu A (2017). A coupled fem/dem model for pipe conveyor systems: Analysis of the contact forces on belt. Powder Technol..

[48] dos Santos e Santos L, Ribeiro Filho PRCF, Macêdo EN (2021). Indentation rolling resistance in pipe conveyor belts: A review. J. Braz. Soc. Mech. Sci. Eng..

[49] Harrison A (1998). Modelling belt tension around a drive drum. Bulk Sol. Handl..

[50] Lodewijks, G. Dynamics of belt systems: Tu delft. *Delft University of Technology* (1996).

[51] Harrison A (1986). Determination of the natural frequencies of transverse vibration for conveyor belts with orthotropic properties. J. Sound Vib..

[52] Harrison, A. Flexural behaviour of tensioned conveyor belts. *Trans. Inst. Eng., Aust., Mech. Eng. (Australia)* (1983).

[53] Kraver TC, Fan GW, Shah JJ (1996). Complex modal analysis of a flat belt pulley system with belt damping and coulomb-damped tensioner. J. Mech. Des..

[54] Xu M (2023). Model-based vibration response analysis and experimental verification of lathe spindle-housing-belt system with rubbing. Mech. Syst. Signal Process..

[55] Kim S-M, Roesset JM (2003). Dynamic response of a beam on a frequency-independent damped elastic foundation to moving load. Can. J. Civ. Eng..

[56] Jafari-Talookolaei R, Kargarnovin M, Ahmadian M, Abedi M (2011). Analytical expressions for frequency and buckling of large amplitude vibration of multilayered composite beams. Adv. Acoust. Vib..

[57] Hop, T. *Vibrations of prestressed beams (in original “Drgania belek sprȩonych”)* (Publishing house of the Silesian University of Technology, 1962).

[58] Weaver W, Timoshenko SP, Young DH (1991). Vibration Problems in Engineering.

[59] Gładysiewicz, L. *Belt conveyors: Theory and calculations (in original “Przenośniki taśmowe: teoria i obliczenia”)* (Publishing house of the Wrocław University of Science and Technology, 2003).

[60] Association, C. E. M. & Conference, C. E. M. A. E. *Belt Conveyors for Bulk Materials: Seventh Edition–Second Printing* (Conveyor Equipment Manufacturers Association (CEMA), 2014).

[61] Shabana AA (1991). Theory of Vibration.

[62] Bortnowski P, Kawalec W, Król R, Ozdoba M (2022). Identification of conveyor belt tension with the use of its transverse vibration frequencies. Measurement.

